# Consumption of Free Sugar Predicts Nutrient Intake of Saudi Children

**DOI:** 10.3389/fnut.2021.782853

**Published:** 2021-11-15

**Authors:** Walaa Abdullah Mumena

**Affiliations:** Clinical Nutrition Department, College of Applied Medical Sciences, Taibah University, Madinah, Saudi Arabia

**Keywords:** free sugar intake, AHA recommendation, nutrient intake, children, Saudi Arabia

## Abstract

Data concerning the association between free sugar intake and nutrient intake in children in the Middle East are not available. This study aimed to explore the association between the consumption of free sugar and nutrient intake among Saudi children. A cross-sectional study included 424 Saudi children ages between 6 and 12 years old and their mothers. An online survey collected sociodemographic data from mothers. Trained data collector personal contacted mothers to arrange for a phone interview in order to collect 24-h dietary recall to assess the dietary intake of children. Only 0.90% of children consumed free sugar within the recent recommendation of the World Health Organization (WHO) of <5% of total energy intake, whereas 10.6% of children consumed free sugar <10% of total energy intake. The percentage of free sugar intake was negatively associated with total energy intake. Multiple linear regression analysis of free sugar consumption and nutrient intake adjusted for children's age and sex indicated that a higher percentage of free sugar intake significantly predicted lower intake of saturated fat, fiber, sodium, potassium, calcium, iron, zinc, and vitamin B_12_. Excessive intake of free sugar predicted a lower intake of many essential nutrients. Interventions that aim to reduce the intake of free sugar are urgently needed in order to improve diet of growing children. Future research to explore top food sources of free sugar is needed to establish effective interventions that aim to limit free sugar intake among children.

## Introduction

Excessive consumption of free sugar has been reported among children in a number of settings. Children in the United Kingdom are consuming on average 13% of their total energy from free sugar (1), while children in Australia are consuming on average 12% of their total energy intake from free sugar (2). The average intake of free sugar among children in the United States is 16% of total energy intake (3). In the Middle East generally and Saudi Arabia specifically, data concerning free sugar consumption among children are lacking. However, high consumption of free sugar is expected, given that multiple studies indicated high consumption of sweets and sugar-sweetened beverages (SSB) by Saudi children (4, 5). Despite the recommendation made by the World Health Organization (WHO), which highlights the need to monitor and limit the consumption of free sugar among all populations (6, 7), data concerning free sugar intake are still unavailable in many settings.

A high intake of free sugar can result in a lower intake of essential micronutrients coming from healthy food options leading to lower quality of diet and consequently increased risk of nutrient insufficiency (8–12). For example, high consumption of free sugar among children was found to be associated with a lower intake of vegetables and fruits (13). In fact, the form of free sugar (solid vs. liquid) can influence the link between the consumption of free sugar and the intake of certain foods. A cross-sectional study conducted among multi-ethnic children reported a negative correlation between consumption of SSB and intake of milk (14). A similar finding was also reported in a Canadian study where a negative association between children's intake of free sugar coming from liquid food sources and dairy products consumption (13). Lower intake of fruits, vegetables, and milk can be an indication of lower quality of diet consumed by children who excessively consume free sugar.

A good quality diet is important, especially for children, to provide important nutrients and maintain good nutritional health and promote growth. Growth impairments may indirectly result from deficiencies of many micronutrients, including zinc, iron, magnesium, and vitamin A (15). Excessive free sugar consumption and lower dietary quality, leading to increased risk of micronutrient deficiencies, also found to be linked to lower school performance (16, 17). In addition, excessive intake of free sugar and lower diet quality are associated with weight gain and obesity (13, 18).

To ensure optimal growth, children should consume adequate quantities of essential nutrients (15). Thus, several health organizations have established a maximum limit of free sugar consumption. The American Heart Association (AHA) and the Saudi Ministry of Health recommended a maximum intake of free sugar of 25 g/day for children, independent of total energy intake (18, 19), whereas the WHO recommended a maximum intake of <10% of total energy intake with recent emphasis on further reduction to <5% of total energy intake coming from free sugar for better health outcomes (6).

In Saudi Arabia, data regarding the association between consumption of free sugar and nutrient intake are lacking. It is critical to assess the intake of free sugar and understand if free sugar consumption predicts the nutrient intake of children. Hence, this study aimed to explore the association between consumption of free sugar and nutrient intake of children in Saudi Arabia.

## Materials and Methods

This cross-sectional study was conducted among Saudi children residing in Saudi Arabia between the ages of 6–12 years old and their mothers. Initial data and consent for participation were collected from 539 using an online survey that was distributed using different social media applications, including WhatsApp, Twitter, and Facebook. Children with chronic diseases or allergies were excluded from this study. We needed a minimum of 365 children to include in this study using 90% power, 99.9% confidence level, two-sided test, and 0.20 effect size (20). The online survey collected data regarding sociodemographics and contact information for mothers. Trained data collector personal contacted mothers to arrange for the phone interview to collect dietary data. Ethical approval was obtained from the ethical committee of the College of Applied Medical Science, Taibah University (certificate: 2020/55/202/CLN).

### Assessment of Dietary Intake

Dietary data were collected during a phone interview with the mother using 24-h dietary recall. One 24-h dietary recall was collected from the full sample, while an additional two recalls were collected from 165 children, as a subsample, to adjust for day-to-day variation. Pictures of standardized food portions and serving sizes of all food groups, drinks, sweets, and candies were sent to mothers during the phone interview to help in estimating the quantities of food consumed by children in the previous 24 h. Mothers provided data regarding their child's dietary intake with the presence of the child and any other person who oversees the child during meals. A random day was collected from children with single recall. For children with multiple recalls, we collected 2 non-consecutive days during the week in addition to 1 day during the weekend.

The Nutritics software version 5.09 (Dublin, Ireland) was used to enter all 24-h dietary recalls. Local food recipes were added to the software after validation of the standardized recipes was completed. If the content of free sugar was not provided in the software, the quantity was estimated based on the nutrition fact label of the food product or the standardized recipe approved by one of the two registered dietitians who oversaw the dietary data collection, entry, and validation.

Nutrient densities for macronutrients and free sugar were calculated as a percent from total energy intake, whereas nutrient densities for micronutrients were calculated as a unit per 1,000 kcal. The WHO recommendation of <5 and <10% of total energy intake coming from free sugar was used to categorize children into two groups: children who consumed free sugar within the recommendation (<5 or <10% of total energy intake coming from free sugar) and children who exceeded the recommendation of free sugar intake (≥5 or ≥10% of total energy coming from free sugar) (6).

### Statistical Analysis

Data of all categorical variables were presented as frequency [percent (%)], while the mean ± standard deviation and median [interquartile range] were used to describe data of continuous variables. The distribution's normality of all continuous variables was assessed using the Shapiro-Wilk test which showed that distributions of dietary intake were mostly skewed. Thus, Mann-Whitney test was used to assess the association between nutrient intake between the two groups of free sugar (<10 and ≥10% of total energy intake). To determine the association between all categorical variables included to describe the characteristics of the study sample across the two groups of free sugar, Fisher's exact test was used. Multiple linear regression analysis was performed to assess nutrient intake that could be predicted by free sugar intake (predictor: percentage of free sugar intake; outcome: nutrient intake) adjusting for child's age and sex. All tests used were two-tailed with a 95% significance level. Bonferroni correction was used to adjust for multiple testing of micronutrient intake in the univariate analysis (15 nutrients: alpha = 0.003). The SPSS version 20 was used to analyze the data presented in this study (SPSS, Inc., Chicago, IL).

## Results

Children and mothers included in this study were 424 after excluding children with missing dietary data (*n* = 79, 14.7%) and children with allergy and chronic diseases (*n* = 36, 6.68%). The median age of children is 9.00 years (7.00–10.00) with 50.5% girls. Ninety-nine percent of children (*n* = 420) exceeded the WHO recommendation of <5% of total energy intake coming from free sugar, whereas 89.4% of children (*n* = 379) exceeded the WHO recommendations of <10% of total energy intake coming from free sugar. Characteristics of children and their mothers were similar across the different groups of free sugar intake (<10 vs. ≥10% of total energy intake) (see Table 1).

**Table 1:** Characteristics of children stratified by percent of free sugar intake.

	**Free sugar intake**	**Free sugar intake**	***p***
	** <10% (*n* = 45)**	**≥10% (*n* = 379)**	
Age, years, mean ± SD	8.98 ± 1.76	8.63 ± 1.86	0.219
median (IQR)	9.00 (7.50–10.00)	8.00 (7.00–10.0)	
**Sex, n (%)**			
Boys	21 (46.7)	189 (49.9)	0.753
Girls	24 (53.3)	190 (50.1)	
**Region of residency**, ***n*** **(%)**			
Western region	21 (46.7)	221 (58.3)	0.295
Central region	10 (22.2)	46 (12.1)	
Eastern region	7 (15.6)	46 (12.1)	
Other regions	7 (15.6)	66 (17.4)	
Maternal age, years, mean ± SD	37.2 ± 6.89	37.1 ± 6.37	0.923
median (IQR)	36.0 (33.5–41.5)	37.0 (32.0–41.0)	
**Maternal education level**, ***n*** **(%)**			
≤ High-school	8 (17.8)	97 (25.6)	0.468
University	31 (68.9)	239 (63.1)	
Postgraduate	6 (13.3)	43 (11.3)	
**Maternal employment status**, ***n*** **(%)**			
Employed	16 (35.6)	157 (41.4)	0.522
Unemployed	29 (64.4)	222 (58.6)	
**Family monthly income**, ***n*** **(%)**			
< SR 4,000	3 (6.70)	26 (6.90)	0.931
SR 4,000–6,000	8 (17.8)	54 (14.2)	
SR 6,001–10,000	10 (22.2)	94 (24.8)	
SR 10,001–15,000	10 (22.2)	98 (25.9)	
>SR 15,000	14 (31.1)	107 (28.2)	
**Maternal weight status**, ***n*** **(%)**			
Underweight	2 (4.40)	12 (3.20)	0.345
Healthy weight	19 (42.2)	144 (38.0)	
Overweight	14 (31.1)	140 (36.9)	
Obese	10 (22.2)	83 (21.9)	

The univariate analysis showed that children the who exceeded the WHO recommendation of <10% of energy coming from free sugar had a lower intake of energy compared to children who consumed free sugar within the recommendation 1,222 kcal/day (1,062–1,466) vs. 1,433 kcal/day (1,179–1,740), respectively; *p* = 0.002). In addition, children who exceeded the WHO recommendation of free sugar had a lower intake of fiber, sodium, and zinc despite their energy intake (*p* < 0.003). Detailed data regarding the nutrient intake of children stratified by the contribution of free sugar to total energy intake are shown in Table 2.

**Table 2:** Nutrient intake of children stratified by percent of free sugar intake.

	**Free sugar intake**	**Free sugar intake**	***p***
	** <10% (*n* = 45)**	**≥10% (*n* = 379)**	
Energy, kcal/day	1,463 ± 352	1,294 ± 344	0.002^*^
	1,433 (1,179–1,740)	1,222 (1,062–1,466)	
Carbohydrate, g/1,000 kcal	49.1 ± 7.22	49.6 ± 7.53	0.632
	49.3 (44.9–52.5)	49.9 (44.9–53.8)	
Protein, g/1,000 kcal	15.1 ± 3.33	15.1 ± 4.13	0.664
	15.3 (12.5–17.9)	14.5 (12.2–17.7)	
Fat, g/1,000 kcal	33.5 ± 6.41	33.4 ± 6.63	0.719
	34.5 (30.0–37.1)	33.4 (28.4–37.8)	
Saturated fat, g/1,000 kcal	29.9 ± 15.1	24.6 ± 16.2	0.003
	29.4 (18.9–37.8)	20.5 (14.0–32.1)	
Trans fat, g/1,000 kcal	6.50 ± 39.8	2.40 ± 34.71	0.019
	0.33 (0.17–1.06)	0.25 (0.06–0.64)	
Fiber, g/1,000 kcal	18.3 ± 11.3	13.9 ± 9.28	0.001^*^
	14.2 (11.0–21.9)	11.7 (16.6)	
Sodium, mg/1,000 kcal	3,919 ± 3,110	2,783 ± 2,575	0.002^*^
	2,885 (1,952–4,873)	2,122 (1,414–3,264)	
Potassium, mg/1,000 kcal	2,352 ± 1,532	1,912 ± 1,142	0.096
	1,848 (1,215–2,992)	1,666 (1,139–2,385)	
Calcium, mg/1,000 kcal	951 ± 493	779 ± 503	0.008
	861 (598–1,176)	662 (443–998)	
Iron, mg/1,000 kcal	14.9 ± 8.18	12.5 ± 7.98	0.029
	12.0 (9.35–21.6)	10.6 (7.44–15.3)	
Zinc, mg/1,000 kcal	8.68 ± 4.97	6.58 ± 4.10	0.002^*^
	7.80 (4.94–9.85)	5.60 (3.82–8.27)	
Vitamin D, μg/1,000 kcal	30.9 ± 47.7	27.5 ± 69.9	0.051
	6.71 (3.57–45.3)	4.87 (2.04–16.5)	
Vitamin C, mg/1,000 kcal	63.3 ± 60.3	73.6 ± 73.19	0.406
	42.4 (15.4–98.1)	51.9 (21.3 (103)	
Vitamin B_12_, μg/1,000 kcal	4.23 ± 8.25	3.06 ± 3.67	0.121
	2.81 (1.81–3.72)	2.41 (1.29–3.67)	

*The numbers presented in the table are mean ± SD and median (IQR).*

* *Alpha was set at 0.003 to denote significance after Bonferroni adjustment*.

Multiple linear regression analysis of free sugar consumption and nutrient intake was performed, adjusting for children's age and sex. Results indicated that a higher percentage of free sugar significantly predicted lower intake of saturated fat [B = −0.21, SE = 0.06 (95% CI: −0.33 to −0.01), R-square = 0.04], fiber [B = −0.14, SE = 0.04 (95% CI: −0.21 to −0.07), R-square = 0.04], sodium [B = −29.8, SE = 9.82 (95% CI: −49.1 to −10.5), R-square = 0.03], potassium [B = −17.3, SE = 4.39 (95% CI: −26.0 to −8.73), R-square = 0.04], calcium [B = −7.84, SE = 1.84 (95% CI: −11.47 to −4.22), R-square = 0.05], iron [B = −0.10, SE = 0.03 (95% CI: −0.16 to −0.05), R-square = 0.04], zinc [B = −0.07, SE = 0.02 (95% CI: −0.10 to −0.04), R-square = 0.06], and vitamin B_12_ [B = −0.03, SE = 0.02 (95% CI: −0.07 to −0.002), R-square = 0.03] (see Table 3).

**Table 3:** Multiple linear regression analysis of the association between children's nutrient intake and percent of free sugar intake.

**Nutrient**	**B**	**SE**	**95% Confidence interval**	***P***	**R-square**
Carbohydrate, %	0.03	0.03	−0.03 to 0.08	0.341	0.01
Protein, %	−0.01	0.02	−0.04 to 0.02	0.667	0.00
Fat, %	−0.02	0.03	−0.07 to 0.02	0.329	0.00
Saturated fat, g/1,000 kcal	−0.21	0.06	−0.33 to −0.01	<0.001^*^	0.04
Trans fat, g/1,000 kcal	−0.08	0.13	−0.34 to 0.18	0.555	0.01
Fiber, g/1,000 kcal	−0.14	0.04	−0.21 to −0.07	<0.001^*^	0.04
Sodium, mg/1,000 kcal	– 29.8	9.82	−49.1 to −10.5	0.003^*^	0.03
Potassium, mg/1,000 kcal	−17.3	4.39	−26.0 to −8.73	<0.001^*^	0.04
Calcium, mg/1,000 kcal	−7.84	1.84	−11.47 to −4.22	<0.001^*^	0.05
Iron, mg/1,000 kcal	−0.10	0.03	−0.16 to −0.05	0.001^*^	0.04
Zinc, mg/1,000 kcal	−0.07	0.02	−0.10 to −0.04	<0.001^*^	0.06
Vitamin D, μg/1,000 kcal	−0.45	0.25	−0.95 to 0.04	0.072	0.03
Vitamin C, mg/1,000 kcal	−0.41	0.27	−0.93 to 0.12	0.128	0.01
Vitamin B_12_, μg/1,000 kcal	−0.03	0.02	−0.07 to −0.002	0.036^*^	0.03

*All models were adjusted for children's age and sex.*

* *Alpha was set at 0.05 to denote significance*.

## Discussion

Only 0.90% of children consumed free sugar within the WHO recommendation of <5% of total energy intake, whereas 10.6% of children consumed free sugar within the WHO recommendation of <10% of total energy intake. Intake of free sugar was inversely associated with energy. A higher intake of free sugar significantly predicted lower intake of saturated fat, fiber, sodium, potassium, calcium, iron, zinc, and vitamin B_12_.

Cardiovascular disease is major health concern in the Gulf countries including Saudi Arabia (21). Excessive free sugar intake has been linked to many cardiovascular diseases, such as cardiac arrhythmias, peripheral vascular disease, hypertension, and coronary artery disease (22). Although it is well-known that post-prandial hyperlipidemia impairs vascular endothelial function, hyperglycemia has been acknowledged as a pro-inflammatory condition and also a primary cause of vascular damage. As such, hyperglycemia is considered an independent risk factor for cardiovascular disease. Therefore, limiting the consumption of free sugar may potentially improve the expression of antioxidant enzymes, reduce the risk of free sugar-induced cardiovascular diseases, and support healthy aging (23).

Previous studies conducted among the Saudi population have mainly focused on the frequency of consumption of SSB, whereas data pertaining total free sugar consumption are lacking (4, 5, 24). For example, a Saudi study conducted in 2015–2016 among 794 school children reported 36.4% of daily consumption of carbonated sugary drinks (5). In the present study, almost all children exceeded the recent WHO recommendation of <5% of total energy intake coming from free sugar, and a large proportion exceeded the <10% recommendation. The proportion of Saudi children exceeded the consumption of free-sugar in our study was higher than the proportions reported among children in the UK and Australia (1, 2). This alarming dietary behavior requires urgent intervention to limit the consumption of free sugar among Saudi children.

Many interventions aim to limit free sugar intake have been implemented in Saudi Arabia. The taxation program on SSB was implemented in 2017 to reduce consumption of SSB and overall intake of free sugar (25). More recently, an intervention program was implemented by the Saudi Food and Drug Authority to increase the awareness concerning free sugar to reduce its consumption among the Saudi population (26). Despite these efforts, free sugar is still excessively consumed by Saudi children. Interactive nutrition education interventions that are delivered face-to-face are proven to be effective in limiting the consumption of free sugar among children and young adults (27, 28). Appropriate interactive nutrition education interventions are needed among children in Saudi Arabia to limit their consumption of free sugar.

It is important to limit the intake of free sugar, as excessive intake is associated with lower diet quality among children and adults (12). High intake of SSB among Saudi children was also found to be associated with poor dietary choices, including consumption of total sugar, iced desserts, savory snacks, and fast food (4). Among Saudi university students, free sugar intake predicted a lower intake of several important nutrients, including fiber, zinc, iron, and vitamin D (29). A study conducted among Canadian children reported an inverse association between free sugar intake and consumption of fruits, vegetables, and dairy products leading to a lower intake of several important micronutrients (13).

Findings of our study show that a higher intake of free sugar significantly predict lower intake of saturated fat, fiber, sodium, potassium, calcium, iron, zinc, and vitamin B_12_. The lower intake of saturated fat, potassium, calcium, zinc, and vitamin B_12_ observed among children with excessive intake of free sugar could be explained by lower consumption of milk and dairy products. A study conducted among multi-ethnic children ages between 3 and 7 years showed a negative correlation between consumption of milk and sweetened beverages (14). Another cross-sectional study conducted among Canadian children found that a higher intake of free sugar from liquid food sources is associated with a lower intake of dairy products (13).

The lower intake of saturated fat, iron, zinc, and vitamin B_12_ predicted by the higher consumption of free sugar could be linked to lower intake of meat among children with excessive intake of free sugar. Total sugar intake has been reported to be negatively correlated with meat consumption (12). Meat is an important source of many nutrients, including protein, zinc, and iron, which are crucial for children's immunity and growth (15, 30).

The lower intake of fiber and potassium predicted by higher consumption of free sugar could be the result of lower consumption of whole-grain products, fruits, and vegetables. Lower intake of fruits and vegetables has been reported among children with a high intake of free sugar (13). Consumption of fruits and vegetables is very low among children and adults in many populations, including Saudi Arabia (31, 32). A notable effort has been made by the WHO and a number of health organizations to promote the consumption of fruits and vegetables to help individuals to meet their minimum requirement of 400 g per day (33). Consumption of sweets, especially between meals, may reduce the opportunity for children to meet their requirement of fruits and vegetables.

The suggested lower consumption of milk and dairy products, meats, fruits, and vegetables are likely to be reflective of the lower quality of diets consumed by children who excessively consume free sugar. Consumption of a balanced diet is important for children to ensure optimal nutritional health, growth, and development (15, 34). Inadequate intake of essential micronutrients may increase the risk for nutritional deficiencies. The prevalence of vitamin D deficiency among Saudi children and adolescences has been estimated to be 14.2 and 50%, respectively (35), whereas the prevalence of anemia in school-aged children was estimated as 11.6% (36).

For a long time, excessive intake of free sugar is thought to be linked to weight gain and obesity. However, recent evidence suggests the link is dependent on total energy intake (37). A study conducted in Saudi Arabia reported a negative association between free sugar intake and weight status, where a high intake of free sugar was linked to lower energy intake, underweight, and lower waist circumference (26). In the present study, children who exceed the recommendation of <10% of energy coming from free sugar consumed significantly lower energy as compared to children whose free sugar intake was within the recommendation. It is possible that children with excessive intake of free sugar seek sugar-rich food due to its accessibility and affordability combined with the limited accessibility to variety of foods, leading to limited overall energy intake. This is further supported by previous research conducted in high-income countries, where the relative price per calorie for sugary foods was found to be cheaper compared to healthy foods (e.g., fruits and vegetables, and milk) (38).

This is the first study to investigate the association between the consumption of free sugar and nutrient intake of children in Saudi Arabia. However, this study is limited by the convenient sampling method used to recruit mothers and their children. Thus, our findings might be limited to children of mothers who are literate and use social media applications.

In conclusion, the excessive intake of free sugar predicted a lower intake of many important nutrients among Saudi children. Inadequate intake of these nutrients may result in micronutrient deficiencies leading to issues related to growth and overall health in the long term. Additional intervention programs to reduce children's intake of free sugar are urgently needed. Future research is also needed to explore food sources of free sugar and investigate predictors of excessive intake of free sugar among children in Saudi Arabia.

## Data Availability Statement

The raw data supporting the conclusions of this article will be made available by the authors, without undue reservation.

## Ethics Statement

The studies involving human participants were reviewed and approved by the Ethical Committee in the College of Applied Medical Science, Taibah University (certificate: 2020/55/202/CLN). Written informed consent to participate in this study was provided by the participants' legal guardian/next of kin.

## Author Contributions

WM conceived the study, conducted the analyses, interpreted the results, wrote the paper, critically reviewed the manuscript, and approved the final version submitted for publication.

## Conflict of Interest

The author declares that the research was conducted in the absence of any commercial or financial relationships that could be construed as a potential conflict of interest.

## Publisher's Note

All claims expressed in this article are solely those of the authors and do not necessarily represent those of their affiliated organizations, or those of the publisher, the editors and the reviewers. Any product that may be evaluated in this article, or claim that may be made by its manufacturer, is not guaranteed or endorsed by the publisher.
